# Comparison of double bundle semitendinosus technique and pedicled quadriceps technique in patellar instability

**DOI:** 10.1002/ksa.12619

**Published:** 2025-02-10

**Authors:** Tayfun Özel, Semih Yaş, Hayati Hürol Türkoğlu, Asim Ahmadov, Muhammet Baybars Ataoğlu, Ulunay Kanatlı

**Affiliations:** ^1^ Department of Orthopedics and Traumatology Ankara Training and Research Hospital Ankara Turkey; ^2^ Department of Orthopedics and Traumatology Health Sciences University Dr. Abdurrahman Yurtaslan Ankara Oncology Education and Research Hospital Ankara Turkey; ^3^ Department of Orthopedics and Traumatology Viranşehir State Hospital Şanlıurfa Turkey; ^4^ Department of Orthopedics and Traumatology Gazi University Hospital Ankara Turkey

**Keywords:** hamstring, medial patellofemoral ligament reconstruction, patellar instability, quadriceps

## Abstract

**Purpose:**

To compare clinical and functional outcomes of medial patellofemoral ligament (MPFL) reconstruction using a minimally invasive pedicled quadriceps tendon (QT) or patella double tunnel technique with semitendinosus tendon (ST) graft in patients with recurrent patella dislocation.

**Methods:**

A retrospective analysis was conducted on 51 patients who underwent MPFL reconstruction between 2014 and 2022, with a minimum 2‐year post‐operative follow‐up. Patients were grouped as QT (*n* = 24) and ST (*n* = 27), alongside a control group of 24 healthy individuals. Kujala, Lysholm and Visual Analogue Scale (VAS) scores were evaluated. Isokinetic tests at 60° angular velocity were performed to calculate the limb symmetry index (LSI) and hamstring/quadriceps (H/Q) ratio.

**Results:**

No significant differences were found between groups regarding age, sex, body mass index, time to surgery, or number of dislocations (n.s). Mean Kujala (QT: 89.2 ± 8.9, ST: 85.4 ± 11.4), Lysholm (QT: 90.6 ± 9.4, ST: 87.9 ± 10.7), and VAS (QT: 0.83 ± 1.3, ST: 0.9 ± 1.1) scores showed no statistically significant differences between the groups (n.s). Extension LSI was significantly higher in QT (92.2 ± 10.0%) than ST (81.4 ± 16.4%, *p* = 0.024), as was flexion LSI (QT: 94.2 ± 10.9%, ST: 83.3 ± 17.5%, *p* < 0.01). H/Q ratios showed no significant differences between operated and non‐operated sides (n.s). No redislocations or patellar fractures occurred. Apprehension signs were positive in two patients (7.4%) in ST and one patient (4.1%) in QT.

**Conclusion:**

MPFL reconstruction with both ST and pedicled QT grafts yields successful results in well‐selected patient groups. Unlike ST, reconstruction with QT results in extension and flexion strength in the operated extremity that is closer to the non‐operated side and to the healthy control group.

**Level of Evidence:**

Level III, retrospective case–control study.

**Abbreviations**
ACLR: anterior cruciate ligament reconstruction
H/Q: hamstring/quadriceps
LSI: limb symmetry index
MPFL: medial patellofemoral ligament
MRI: magnetic resonance imaging
PFI: patellofemoral instability
QT: quadriceps tendon
ROM: range of motion
ST: Semitendinosus Tendon
VAS: Visual Analogue Scale

## INTRODUCTION

Patellofemoral instability (PFI) is a symptomatic and multifactorial problem often characterized by the patella's misaligned movement within the trochlear groove during knee flexion between 0° and 30° [1]. The average incidence of patellar dislocations is 5.8 per 100,000 in the general population, while it rises to 29 per 100,000 in the 10–17 age group. The majority of patients are young (10–16 years) and female [18, 22, 29].

The medial patellofemoral ligament (MPFL) contributes approximately 60% to preventing lateral displacement of the patella during knee flexion between 0° and 30° [9, 49]. Magnetic resonance imaging (MRI) studies have shown that the MPFL is damaged in 96%–100% of cases following an initial dislocation [45]. The likelihood of recurrent dislocations in conservatively treated cases ranges from 15% to 44%. If a patient experiences a second dislocation, the risk of subsequent dislocation episodes increases to 50% [8]. Since untreated patellar dislocations can lead to recurrent dislocations, chronic knee pain, and osteoarthritis, appropriate treatment is essential.

Numerous bone and soft tissue surgical procedures have been described for the surgical treatment of PFI. These procedures can be performed individually or in combination. MPFL reconstruction is the most commonly preferred option among them and has the lowest redislocation rate [24, 58]. In the literature, MPFL reconstructions have been described using various grafts, such as semitendinosus, gracilis, quadriceps, patellar tendon and allografts, with different femoral and patellar fixation methods [10, 11]. One of the most widely used techniques involves a semitendinosus tendon (ST) graft with the double tunnel technique [32]. Recently, reconstruction using a pedicled superficial quadriceps tendon (QT) graft has gained popularity due to its advantages, such as eliminating the need for tunnels or implants in the patella and its minimally invasive approach [16]. Few studies have compared the clinical outcomes and complications of these two grafts using different patellar fixation techniques, finding no significant differences. However, no studies in the literature have evaluated flexion and extension strength outcomes for these techniques [53, 54].

The aim of this study is to compare clinical and functional outcomes of minimally invasive MPFL reconstruction using a pedicled QT graft and the double tunnel technique with an ST graft with at least 2‐year follow‐up in patients with recurrent patellar dislocation. The secondary aim was to compare patients with patellar instability to non‐injured control group. The hypothesis of this study was that there would be no difference in clinical and functional outcomes between MPFL reconstruction with the pedicled QT technique and the double bundle ST technique.

## MATERIALS AND METHODS

The approval for this study was obtained from the Gazi University Ethics Committee under the research code 2024‐43. Informed consent was obtained from all participants prior to the study.

This study retrospectively analyzed the clinical outcomes of patients diagnosed with PFI who underwent MPFL reconstruction using two different techniques performed by two senior surgeons at the Gazi University Faculty of Medicine, Department of Orthopedics and Traumatology, between 2014 and 2022. All patients had a minimum follow‐up period of at least 2 years. PFI was defined as either having at least two patellar dislocations or experiencing a single patellar dislocation with persistent instability symptoms (pain, subluxation or both) despite conservative treatment, supported by clinical and radiological evidence of MPFL rupture. All patients underwent imaging studies, including radiographs (AP, lateral and Merchant views), computed tomography and MRI.

**Figure 1:**
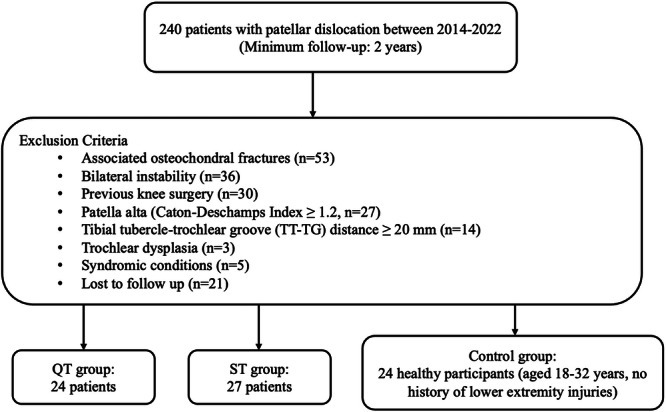
Flowchart of patient selection procedure. QT, quadriceps tendon; ST, semitendinosus tendon.

Between 2014 and January 2022, a total of 240 patients underwent surgery for patellar dislocation. Patients who underwent only MPFL reconstruction without any additional procedures were included in this study. Exclusion criteria were as follows: osteochondral fractures (*n* = 53), bilateral instability (*n* = 36), previous knee surgery (*n* = 30), patella alta defined as Caton–Deschamps Index ≥1.2 (*n* = 27), tibial tubercle‐trochlear groove distance ≥20 mm (*n* = 14), trochlear dysplasia (*n* = 3) and syndromic conditions (*n* = 5). Additionally, 21 patients were lost to follow‐up (Figure 1).

The remaining 51 patients were included in the study, divided into two groups: QT (*n* = 24) and ST (*n* = 27). The control group consisted of 24 healthy participants aged 18–32 years who had no history of lower extremity injuries, such as meniscal, ligamentous or chondral injuries (Table 1).

**Table 1:** Demographic characteristics of the participants.

	Control (*n* = 24)	QT (*n* = 24)	ST (n = 27)	*p* **value**	Effect size
Age, years	23.1 ± 3.3	22.0 ± 5.8	23.9 ± 6.8	n.s	0.049
Male/female	13/11	11/13	12/15	n.s	0.085
Body mass index	23.0 ± 3.3	25 ± 5.7	24.5 ± 4.5	n.s	0.007
Dominant side (R/L)	23/1	23/1	25/2	n.s	0.069
Operated side (R/L)		15/9	13/14	n.s	0.141
Follow‐up, months		31.1 ± 7.5	45.1 ± 21.9	*p* < 0.01	0.16
Number of dislocations		7.3 ± 10.4	5.4 ± 8.0	n.s	0.045
Time to operation, months		44.5 ± 52.9	28.4 ± 30.0	n.s	0.021

*Note*: Data presented as mean ± standard deviation; *p* = 0.05. Effect sizes for the Kruskal–Wallis test were calculated using epsilon‐squared (*ε*²), and Cramér's *V* was used for chi‐square tests. Epsilon‐squared values are interpreted as small (<0.01), medium (0.01–0.08) and large (>0.08). Cramér's *V* values are interpreted as small (0.10–0.29), medium (0.30–0.49) and large (≥0.50).

Abbreviations: QT, quadriceps tendon autograft; ST, semitendinosus tendon autograft.

For the control group, LSI was calculated as $\mathrm{LSI}=\left(\frac{\mathrm{Maximum}\;\mathrm{torque}\;\mathrm{for}\;\mathrm{the}\;\mathrm{non}\;\mathrm{dominant}\;\mathrm{side}}{\mathrm{Maximum}\;\mathrm{torque}\;\mathrm{for}\;\mathrm{the}\;\mathrm{dominant}\;\mathrm{side}}\right)\times 100\%$.

The evaluated parameters included demographic and baseline data (age, sex, body mass index, dominant side, operated side, follow‐up period, number of dislocations and time to operation), as well as clinical and functional outcomes. The latter consisted of Kujala, Lysholm and VAS scores, ROM for extension and flexion, isokinetic strength (LSI at 60° angular velocity) and muscle balance (H/Q ratios for both operated and non‐operated sides).

### Surgical technique

The pedicled QT technique was performed by a senior orthopaedic surgeon (UK), and the patellar double tunnel technique was performed by another senior orthopaedic surgeon (MBA). After administering general or regional anaesthesia, the patient was placed in a supine position, and the diagnosis was reconfirmed through a physical examination. Prophylactic antibiotics were administered, followed by the application of a tourniquet to the thigh. After proper surgical site preparation and draping, the affected knee was positioned appropriately. All patients underwent diagnostic arthroscopy, and any associated intra‐articular pathologies were documented.

### Double tunnel with ST tendon graft technique

The ipsilateral ST was harvested according to the standard procedure, and both ends of the tendon were sutured using a 2.0 absorbable suture [5]. A longitudinal incision, approximately 1.5 cm on the lateral side and 3–4 cm on the medial side of the patella, was made to include the proximal half of the patella.

Under fluoroscopic guidance, two parallel K‐wires were inserted into the upper two‐thirds of the patella, ensuring no damage to the chondral structures, with a spacing of 10–15 mm between them. Over these K‐wires, tunnels slightly larger than the graft diameter (by 1 mm) were drilled. Subsequently, a 2–3 cm longitudinal incision was made from the medial epicondyle to the adductor tubercle. Using fluoroscopic guidance, a guidewire was inserted referencing the Schöttle point [56]. A bone tunnel with a diameter of 1 mm greater than that of the graft was created through the guide wire.

**Figure 2:**
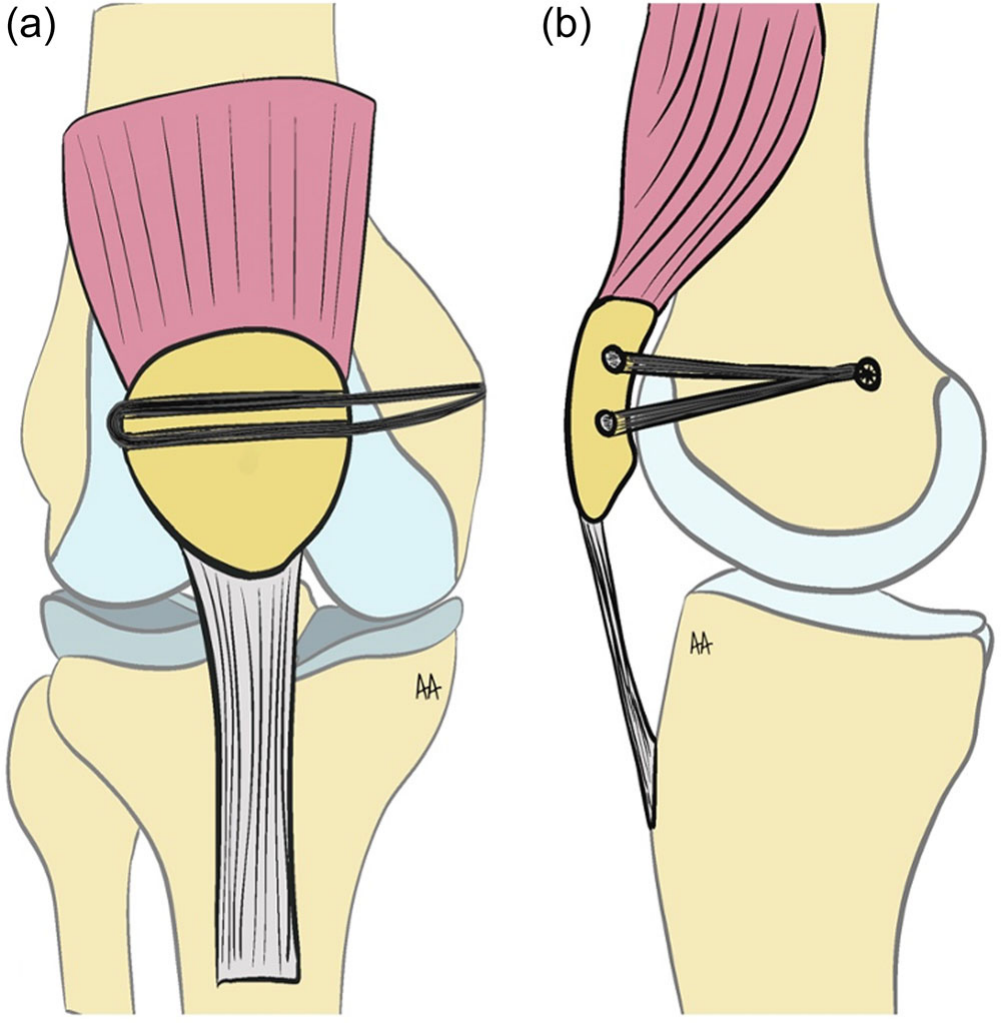
Illustration of double bundle semitendinosus technique. (a) Anteroposterior view. (b) Sagittal view.

The tendon graft was passed through the patellar tunnels to form a loop on the lateral side of the patella [6]. Both ends of the graft were transferred to the femoral side through the medial retinaculum and joint capsule using a suture shuttle. After positioning the graft into the femoral tunnel via the shuttle, the graft was tensioned appropriately, and the knee was cycled through flexion and extension five times to ensure proper tensioning. Once the appropriate tension was achieved, the lateral edge of the patella was aligned with the lateral trochlear groove, and femoral fixation was achieved using a 6–8 × 25 mm^2^ interference screw (Figure 2).

### Reconstruction with pedicled QT graft

**Figure 3:**
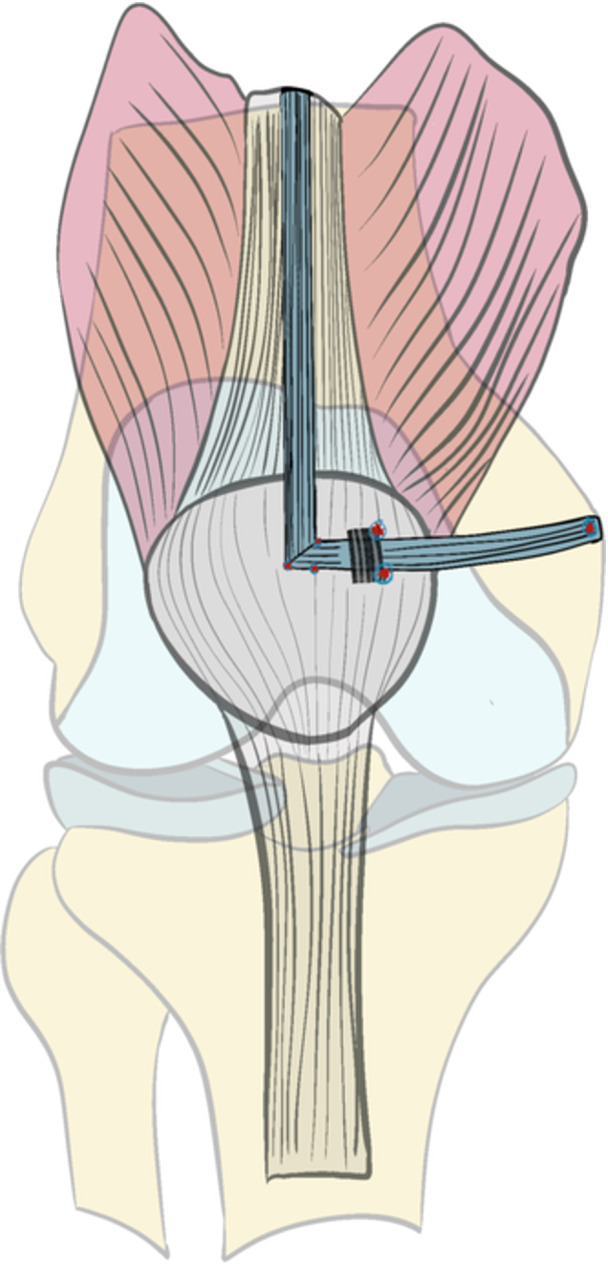
Illustration of pedicled quadriceps technique.

As described by Fink et al. [16], a 2.5–3 cm transverse incision was made at the superomedial aspect of the patella, following Langer's lines. The prepatellar bursa was incised longitudinally to expose the QT. Using a double‐edged knife (KARL STORZ), a graft measuring 1 cm in width, 8 cm in length and 3 mm in thickness was harvested from the midline of the superior patella, ensuring its distal attachment remained intact. The distal incision line of the graft was extended on the anterior surface of the patella using a surgical knife, measuring 2 cm laterally and 1 cm medially. After suturing the free end of the graft with 2‐0 absorbable sutures, it was rotated 90° medially and passed under the prepatellar tissue. Additional absorbable sutures were placed at the medial patellar border and the lateral edge of the tendon incision to secure the graft. Subsequently, graft was transferred to the femoral side through the medial retinaculum and joint capsule using a suture shuttle. Femoral fixation was achieved in the same manner as described for the ST graft technique (Figure 3).

### Post‐operative management

The same rehabilitation programme was applied to both groups post‐operatively. On the first post‐operative day, straight leg‐raising exercises were recommended to strengthen the quadriceps muscles. Patients were instructed to use an adjustable locking knee brace with a range of motion (ROM) limited to 0°–90° for 6 weeks, during which passive ROM exercises were initiated. Weight‐bearing was permitted with the knee in full extension, as tolerated by the patient. Functional activities such as walking, jogging, and running were allowed after three months, once full joint ROM and strength were achieved. Return to contact sports was permitted 4–6 months post‐operatively.

### Outcome evaluations

Post‐operatively, patients were followed up every 2 weeks for the first 6 weeks, every three months during the first year, and annually thereafter. Evaluations were conducted during the final follow‐up. Data recorded included the patient's name, age, gender, height, weight, dominant side, operated side, total number of dislocations, time from the initial dislocation to surgery and the number of redislocations post‐surgery. Special tests specific to patellar dislocation, including the patellar apprehension test and patellar glide test, were performed and documented. Post‐operative complications, such as new dislocations or patellar fractures, were recorded. Clinical and functional outcomes were assessed using the Lysholm, Kujala and VAS scores [7, 30, 36].

### Isokinetic strength test

To evaluate the isokinetic knee strength performance of the patients, data were collected at their final follow‐up using the Isomed 2000 dynamometer (D&R Ferstl GmbH). Before testing, each patient's current height and weight were measured, and the data were calibrated according to their body mass index. A 15‐min warm‐up session was conducted for all patients before the test.

Muscle strength was assessed using knee flexion and extension tests at 60°/s, with two trial repetitions followed by six‐test repetitions. Testing began with the non‐operated side, and measurements were conducted separately for each knee. During the tests, patients were seated on the dynamometer with stabilization provided at the shoulders, hips and legs. Verbal encouragement was given to motivate the participants. All isokinetic variables were gravity‐adjusted, and the dynamometer was calibrated before and after each test according to the manufacturer's instructions. All calibrations and tests were performed by the same operator.

To normalize strength results among participants, the limb symmetry index (LSI) and hamstring‐to‐quadriceps (H/Q) ratio were calculated using the maximum torque for each side.

The LSI was calculated as the percentage of the operated side's strength relative to the non‐operated side's strength $\mathrm{LSI}=\left(\frac{\mathrm{Maximum}\;\mathrm{torque}\;\mathrm{for}\;\mathrm{the}\;\mathrm{operated}\;\mathrm{side}}{\mathrm{Maximum}\;\mathrm{torque}\;\mathrm{for}\;\mathrm{the}\;\mathrm{non}-\mathrm{operated}\;\mathrm{side}}\right)\times 100\%$. Values below 100% indicated a strength deficit in the reconstructed limb, while values above 100% indicated greater strength in the reconstructed limb. Common clinical criteria for quadriceps strength evaluation suggest that an LSI ≥ 80% is required to begin running, and an LSI ≥ 90% is necessary for returning to sports [23, 44].

For the control group, LSI was calculated as $\mathrm{LSI}=\left(\frac{\mathrm{Maximum}\;\mathrm{torque}\;\mathrm{for}\;\mathrm{the}\;\mathrm{non}-\mathrm{dominant}\;\mathrm{side}}{\mathrm{Maximum}\;\mathrm{torque}\;\mathrm{for}\;\mathrm{the}\;\mathrm{dominant}\;\mathrm{side}}\right)\times 100\%$. It has been mentioned in the studies that maximum muscle strength does not show a significant difference between the dominant and non‐dominant sides [2, 59].

The H/Q ratio for both operated and control groups was calculated separately for each limb using the formula $H/Q\;\mathrm{ratio}=\left(\frac{\mathrm{Maximum}\;\mathrm{torque}\;\mathrm{for}\;\mathrm{hamstrings}}{\mathrm{Maximum}\;\mathrm{torque}\;\mathrm{for}\;\mathrm{quadriceps}}\right)\times 100\%$ [57].

### Statistical analysis

A power analysis was conducted using G*Power (version 3.1) to estimate the sample size and the study's statistical power [13]. The total sample size was determined to be 44, with parameters set at an alpha level of 0.05, a power of 0.80 and an effect size (dz) of 0.8 [53, 60]. Data analysis was performed using SPSS version 28 (IBM SPSS Statistics, Version 28.0, Armonk, NY: IBM Corp). Descriptive statistics were calculated for all variables. The Shapiro–Wilk test was used to assess the normality of distribution for each group. Due to non‐normal distribution of the data, non‐parametric tests were applied for statistical analysis.

The Kruskal–Wallis test was employed to compare the three groups (semitendinosus graft, quadriceps graft and control) in terms of the variables of interest. Post hoc analyses were performed using the Mann–Whitney *U* test. Post hoc pairwise comparisons following significant Kruskal–Wallis tests were conducted using the Dunn–Bonferroni method to control for Type I error. For all pairwise comparisons, *p* values were adjusted using the Bonferroni–Holm correction for multiple comparisons. Statistical significance was set at *p* < 0.05.

To evaluate symmetry in the distribution of the H/Q ratio between the operated and non‐operated sides, the Wilcoxon signed‐rank test was applied. Analyses were conducted separately for the ST, QT and control groups. A *p* value of <0.05 was considered statistically significant.

The relationship between the follow‐up periods of the patients and their functional scores and flexion‐extension strengths was evaluated using Sperman's correlation analysis.

Effect sizes were calculated to enhance the interpretability of results. For the Kruskal–Wallis test, epsilon‐squared (*ε*²) was used, while Cramér's *V* was calculated for chi‐square tests. Effect sizes for the Wilcoxon and Mann–Whitney *U* test were calculated using rank‐biserial correlation (*r*).

## RESULTS

The study evaluated 24 patients who underwent reconstruction with a QT graft, 27 patients who underwent reconstruction with an ST graft, and 24 healthy individuals as the control group, all after a minimum follow‐up period of 24 months. The demographic data of the patients are presented in Table 1.

There was a statistically significant difference in follow‐up times between the two groups (*p* < 0.01). A Spearman's rank correlation test was conducted to assess the relationship between the follow‐up period and the clinical scores (Kujala, Lysholm, VAS, LIS60 extension and LIS60 flexion). The results showed no statistically significant correlations between follow‐up duration and any of the scores (all *p* values n.s).

### Clinical results

At the final clinical evaluation, all patients demonstrated full active and passive ROM in the affected joint. The mean Lysholm scores (QT: 90.9 ± 9.4, ST: 87.9 ± 10.7), Kujala scores (QT: 89.2 ± 8.9, ST: 85.4 ± 11.4) and VAS scores (QT: 0.8 ± 1.3, ST: 0.9 ± 1.1) showed no statistically significant differences between the groups (n.s.). The detailed results are presented in Table 2.

**Table 2:** Comparison of post‐operative clinical scores.

	QT (*n* = 24)	ST (*n* = 27)	*p* **value**	Effect size
VAS for pain	0.8 ± 1.3	0.9 ± 1.1	n.s	−0.068
Lysholm score	90.6 ± 9.4	87.9 ± 10.7	n.s	−0.13
Kujala score	89.2 ± 8.9	85.4 ± 11.4	n.s	−0.155

*Note*: Data presented as mean ± standard deviation; *p* = 0.05. Effect sizes for the Mann–Whitney *U* test were calculated using rank‐biserial correlation (*r*). This measure quantifies the strength of the relationship between groups. Values of rare were interpreted as small (0.1 ≤ *r* < 0.3), medium (0.3 ≤ *r* < 0.5) and large (*r* ≥ 0.5).

Abbreviations: QT, quadriceps tendon autograft; ST, semitendinosus tendon autograft; VAS, Visual Analogue Scale.

## FUNCTIONAL RESULTS

At the final follow‐up, interlimb strength differences in the study and control groups were assessed using isokinetic dynamometric strength tests at 60° extension and 60° flexion. Based on the measurements, the LSI and H/Q values were calculated. The LSI values at 60° extension were 92.2 ± 10.0% in the QT group, 81.4 ± 16.4% in the ST group and 93.4 ± 13.8% in the control group. At 60° flexion, the LSI values were 94.2 ± 10.9% in the QT group, 83.3 ± 17.5% in the ST group and 96.9 ± 9.0% in the control group. The LSI values in the QT group were significantly higher than those in the ST group for both flexion (*ε*² = 0.240, 95% CI = 0.129–0.352, *p* < 0.05) and extension (ε² = 0.089, 95% CI = 0.021–0.157, p < 0.05). However, no statistically significant difference was found between the QT and control groups (n.s.) (Table 3).

**Table 3:** Comparison of isokinetic dynamometric strength test results.

	Control (*n* = 24)	QT (*n* = 24)	ST (*n* = 27)	*p* **value**	Effect size
LSI 60° extension (%)	93.4 ± 13.8	92.2 ± 10.0	81.4 ± 16.4	*p* < 0.05	0.089
LSI 60° flexion (%)	96.9 ± 9.02	94.2 ± 10.9	83.3 ± 17.5	*p* < 0.01	0.24
Operated side H/Q (%)	66.8 ± 10.5	75.2 ± 12.4	69.9 ± 16.8	n.s	0.057
Non‐operated side H/Q (%)	64.8 ± 8.6	74.1 ± 12.9	70.0 ± 15.6	n.s	0.063

*Note*: Data presented as mean ± standard deviation. *p* = 0.05. Effect sizes for the Kruskal–Wallis test were calculated using epsilon‐squared (*ε*²). For the control group, LSI was calculated as $\mathrm{LSI}=\left(\frac{\mathrm{Maximum}\;\mathrm{torque}\;\mathrm{for}\;\mathrm{the}\;\mathrm{non}-\mathrm{dominant}\;\mathrm{side}}{\mathrm{Maximum}\;\mathrm{torque}\;\mathrm{for}\;\mathrm{the}\;\mathrm{dominant}\;\mathrm{side}}\right)\times 100\%$.

Abbreviations: H/Q, hamstring/quadriceps; LSI, limb symmetry index; QT, quadriceps tendon autograft; ST, semitendinosus tendon autograft.

The H/Q ratios of the operated side in the patient groups and the dominant side in the control group were 76.6 ± 14.0% in the QT group, 69.9 ± 16.8% in the ST group and 66.8 ± 10.5% in the control group. Similarly, the H/Q ratios of the non‐operated side in the patient groups and the non‐dominant side in the control group were 74.1 ± 12.9% in the QT group, 70.0 ± 15.6% in the ST group and 64.8 ± 8.6% in the control groups. No statistically significant differences were found in the H/Q ratios among the groups (n.s.) (Table 3). Additionally, when the operated and non‐operated sides within each of the three groups were compared to their contralateral extremities, no statistically significant differences were observed (n.s.) (Table 4).

**Table 4:** Comparison of the H/Q ratio between the operated side and the contralateral side.

		Mean ± SD	Median	Interquartile range	*p* **value**	Effect size
ST	Operated side H/Q (%)	69.9 ± 16.8	65.7	22.9	n.s	−0.153
	Non‐operated side H/Q (%)	70.0 ± 15.6	70.3	20.4		
QT	Operated side H/Q (%)	75.2 ± 12.4	74.0	23.2	n.s	−0.341
	Non‐operated side H/Q (%)	74.1 ± 12.9	73.5	24.1		
Control	Dominant side H/Q (%)	66.8 ± 10.5	68	16.4	n.s	−0.227
	Non‐dominant side H/Q (%)	64.8 ± 8.6	63.8	14.1		

*Note*: *p* = 0.05. Effect sizes for the Wilcoxon signed‐rank test were calculated using rank‐biserial correlation (*r*).

Abbreviations: H/Q, hamstring/quadriceps; QT, quadriceps tendon autograft; ST, semitendinosus tendon autograft.

## DISCUSSION

The most significant finding of this study is that patients who underwent MPFL reconstruction using a pedicled QT graft demonstrated significantly better flexion and extension strength compared to those who underwent patellar double tunnel MPFL reconstruction with an ST graft. Additionally, the flexion and extension strength in the QT group were comparable to the results observed in the healthy control group. To the best of our knowledge, no previous study in the literature has performed isokinetic strength analysis following MPFL reconstruction using a pedicled QT graft. This study is the first to conduct isokinetic strength analysis after minimally invasive MPFL reconstruction with a pedicled QT graft and to compare the results with those of the ST double tunnel group and the control group.

In this study, no significant differences were observed between the HT and QT groups in preoperative demographic data, post‐operative VAS, Kujala and Lysholm functional scores, or joint ROM. Both groups achieved satisfactory outcomes, consistent with the literature. A study comparing QT and HT graft reconstructions reported post‐operative Kujala scores of 88.4 ± 5 and 89.4 ± 10.2, respectively, and post‐operative Lysholm scores of 88.9 ± 10.1 and 84.8 ± 12.9, respectively [53]. Another study found post‐operative Kujala scores of 94.9 ± 4.1 and 94.2 ± 8.0 for QT and HT grafts, respectively, and Lysholm scores of 94.1 ± 5.0 and 93.2 ± 7.0, respectively, concluding that both techniques yielded satisfactory results [54]. Systematic reviews and meta‐analyses have reported mean post‐operative Kujala scores of 84.4–94 for QT graft reconstructions and 81.7–96.4 for HT grafts [40, 41]. These findings suggest that both techniques similarly improve knee function.

An LSI of <90% is considered pathological, indicating strength loss and an increased risk of additional injury during the return to sports [23, 43, 55]. Another study mentioned that to begin effective activities, an individual must have trace or no effusion, full, symmetrical knee ROM, and greater than 80% quadriceps LSI [31]. Ercan et al. [12], in their study with 80 patients followed for an average of 40 months, reported flexor and extensor strength losses in both single‐tunnel and double‐tunnel MPFL reconstructions performed with ST grafts. Similarly, Ronga et al. [51], in their study of 28 patients with an average follow‐up of 3 years, observed that all isokinetic parameters of the operated limb were lower than those of the non‐operated side following MPFL reconstruction using the ST double tunnel technique. Other studies in the literature have reported strength losses of up to 20% [39, 46]. In our study, we observed strength losses of up to 20% in the LSI values for both extension and flexion in the ST group. Conversely, in the QT group, the LSI values for both extension and flexion were >90%, indicating results comparable to those of the control group, with no flexion or extension strength loss.

The only study measuring extensor strength after MPFL reconstruction with a QT graft was conducted by Rhatomy et al. [50], who evaluated quadriceps strength using a hand dynamometer in 21 patients at 6 months post‐operatively. These patients underwent MPFL reconstruction combined with lateral release. According to their findings, no difference in quadriceps strength was observed between the operated and non‐operated sides. Although their study differs from ours in being non‐isokinetic, involving lateral release, and having a shorter follow‐up period, the absence of extensor strength loss in their results supports the findings of our study.

Since no other studies have performed isokinetic strength analysis following MPFL reconstruction with QT, we reviewed studies on anterior cruciate ligament reconstruction (ACLR), another major procedure utilizing QT. In ACLR, the QT is harvested from the patella either with or without a bone block and as a full‐thickness or partial‐thickness graft [19]. Studies have reported approximately a 20% loss in isokinetic extensor strength compared to the contralateral side following ACLR with QT harvested with a bone block [20, 33, 57]. Iriuchishima et al. [25] found an average LSI value of 85.1% at 1 year post‐operatively in patients undergoing ACLR with a full‐thickness QT graft harvested without a bone block. Similarly, Martin‐Alguacil et al. [38] reported better isokinetic results in the QT group compared to the HT group at 1 year post‐operatively in ACLR performed without a bone block. Letter et al. [35], in a study comparing partial and full‐thickness QT grafts for ACLR, observed no loss in extension strength in the operated limb compared to the contralateral side in the partial graft group. They also noted faster recovery and earlier return to sports with partial‐thickness grafts compared to full‐thickness grafts. In ACLR, the tendon thickness of a full‐thickness QT graft is reported to average 7 mm (range: 6.4–7.8 mm) [21], whereas the tendon thickness used in our MPFL reconstruction was 3 mm. We believe this reduced graft thickness could be one of the reasons for the absence of strength loss observed in MPFL reconstruction with QT in our study. This hypothesis is supported by Parrino et al. [48], who conducted isokinetic extension tests and electromyography in ACLR performed with partial and full‐thickness QT grafts. They found that full‐thickness grafts disrupted the musculotendinous junction, leading to reduced electromyographic delay and impaired maximal extension strength. Conversely, partial‐thickness grafts preserved electromyographic delay and maximal extension strength at levels comparable to those of healthy knees.

The H/Q ratio, which represents the balance between the agonist and antagonist muscles surrounding the knee joint, plays a crucial role in ensuring a safe return to full activity and is also used as a screening tool to minimize the risk of sports injuries [3]. This ratio is particularly helpful during rehabilitation following unilateral injuries, as it allows treatment to be tailored using the healthy limb as a reference [37]. In healthy individuals, the H/Q ratio at the knee varies between 50% and 80%, depending on the degree of knee flexion and the angular velocity of measurement. As the ratio approaches 100%, the functional capacity of the hamstrings in stabilizing the knee increases, thereby reducing the likelihood of knee injuries [17, 28, 52]. Another perspective suggests that achieving symmetry between the H/Q ratios of the injured and healthy extremities is ideal [27]. In our study, the H/Q ratios were similar between the injured and healthy extremities in all three groups, with all values within normal limits. The highest H/Q ratio was observed in the QT group. To date, no studies in the literature have evaluated the H/Q ratio following MPFL reconstruction. However, studies comparing H/Q ratios after ACLR using QT and HT grafts have reported better H/Q ratios in the QT group [4, 17, 38].

Numerous techniques for MPFL reconstruction have been described in the literature; however, the question of which technique is the most effective remains a subject of debate. Mohammed et al. [42] argued that double‐bundle reconstruction better mimics the fan‐shaped anatomical structure of the MPFL compared to single‐tunnel reconstruction. While this technique eliminates the need for additional materials such as anchors or endobuttons by creating double tunnels in the patella, it carries a risk of serious complications, such as patellar fractures.

In the minimally invasive QT technique, the use of tunnels and anchors is avoided, thereby reducing the risk of iatrogenic patellar fractures or anchor malposition [15, 16]. The 3‐mm thick and 10‐mm wide QT graft has been reported to closely resemble the natural MPFL in terms of biomechanical properties, including maximum load capacity and stiffness. In contrast, the ST graft has been shown to be three times stiffer, making it more prone to over‐tensioning and malposition, potentially increasing compressive forces on the patellofemoral joint and restricting motion [14, 34].

Jackson et al. [26], in a systematic review of 28 studies involving a total of 1478 patients, reported that complications following MPFL reconstruction ranged from 0% to 32.3%, with failure rates between 0% and 10.7%. Major complications included patellar fractures (0%–8.3%), recurrent patellar instability (0%–18.8%) and post‐operative joint stiffness or restricted ROM (0%–20%).

In our study, no cases of patellar fractures or redislocations were observed in either group. However, positive apprehension signs were detected in two patients (7.4%) in the HT group and in one patient (4.1%) in the QT group. Runer et al. [53] reported a 12.5% rate of positive patellar instability findings in the HT group and 6.3% in the QT group, with both groups showing a 3.1% rate of positive apprehension signs.

## LIMITATIONS

This study has several limitations. First, as patients were evaluated retrospectively, preoperative functional scores and isokinetic measurements were not available. This limitation prevents us from determining whether the preoperative functional status of the patients influenced surgical success and the post‐operative recovery of flexor and extensor strength. However, it is also acknowledged that performing preoperative isokinetic measurements on a knee with trauma‐induced damage and swelling could potentially harm the patient and yield inaccurate strength analyses due to quadriceps inhibition caused by oedema [47].

Another limitation is that the surgeries were performed by two different surgeons, which might have introduced variability in surgical technique. The selection of graft was determined by the preferences of the surgeon and the patient, resulting in selection bias. Additionally, the small sample size and relatively short follow‐up period are other limitations of this study. Longer follow‐up studies on this subject may provide more accurate results regarding the reliability of both techniques.

## CONCLUSION

In the treatment of patellar instability, MPFL reconstruction with a pedicled QT graft and the patellar double tunnel technique using an ST graft demonstrated similar functional scores after a minimum follow‐up of 2 years. However, extension and flexion strength were higher in the QT group compared to the ST group following MPFL reconstruction.

## AUTHOR CONTRIBUTIONS

**Tayfun Özel**: Study design; literature review; data collection; manuscript writing; manuscript editing. **Semih Yaş**: Literature review; manuscript writing; manuscript editing. **Hayati Hürol Türkoğlu**: Data collection; statistical analysis; manuscript editing. **Asim Ahmadov**: Statistical analysis; data collection; manuscript editing. **Muhammet Baybars Ataoğlu**: Study design; supervision; manuscript writing; manuscript editing. **Ulunay Kanatlı**: Study design; supervision; manuscript writing; manuscript editing. All authors read and approved the final manuscript.

## CONFLICT OF INTEREST STATEMENT

The authors declare no conflicts of interest.

## ETHICS STATEMENT

The study was approved by the Ethics Committee of Gazi University (2024‐43). All participants consented to participate in the study.

## Data Availability

The data that support the findings of this study are available on request from the corresponding author.
